# Overview of Phlorotannins’ Constituents in Fucales

**DOI:** 10.3390/md20120754

**Published:** 2022-11-30

**Authors:** Marcelo D. Catarino, Sónia M. G. Pires, Sónia Silva, Filipa Costa, Susana S. Braga, Diana C. G. A. Pinto, Artur M. S. Silva, Susana M. Cardoso

**Affiliations:** 1 LAQV-REQUIMTE, Department of Chemistry, University of Aveiro, 3810-193 Aveiro, Portugal; 2 School of Engineering, University of Minho, Campus of Azurém, 4800-058 Guimarães, Portugal

**Keywords:** Phaeophyceae, brown algae, sargassaceae, fucaceae, phlorotannins, phenolic compounds, structural elucidation, mass spectrometry, NMR

## Abstract

Fucales are an order within the Phaeophyceae that include most of the common littoral seaweeds in temperate and subtropical coastal regions. Many species of this order have long been a part of human culture with applications as food, feedand remedies in folk medicine. Apart from their high nutritional value, these seaweeds are also a well-known reservoir of multiple bioactive compounds with great industrial interest. Among them, phlorotannins, a unique and diverse class of brown algae-exclusive phenolics, have gathered much attention during the last few years due to their numerous potential health benefits. However, due to their complex structural features, combined with the scarcity of standards, it poses a great challenge to the identification and characterization of these compounds, at least with the technology currently available. Nevertheless, much effort has been taken towards the elucidation of the structural features of phlorotannins, which have resulted in relevant insights into the chemistry of these compounds. In this context, this review addresses the major contributions and technological advances in the field of phlorotannins extraction and characterization, with a particular focus on Fucales.

## 1. Introduction

Fucales is one of the largest and most diverse orders of the Phaeophyceae class (i.e., brown algae), which includes some of the most common seaweeds in temperate and subtropical coastal regions, from seven distinct families, namely Sargassaceae, Fucaceae, Himanthaliaceae, Durvillaeaceae, Notheiaceae, Hormosiraceae, and Seirococcaceae [1]. The members of this order are multicellular and have the typical structure of large marine macroalgae, with a well-differentiated rhizoid, stipe, and lamina [2]. The blade is usually enlarged and may have gas vesicles to float freely in the water. 

Sargassaceae is the major representative family of Fucales, with *Sargassum*-genus representing about 65% of its members. In Europe, native-Sargassaceae species, such as *Cystoseira*, *Ericaria*, and *Gongolaria*, occupy mostly the intertidal rockpools and/or the subtidal region. Particularly, *Cystoseira*, accounting for more than 30 species, is one of the most important genera found in the Mediterranean Sea and Atlantic Ocean, essential for biodiversity and ecosystem functioning [3]. It is also claimed as a promising source of bioactive compounds [4]. Because of their location, species from this genus are exposed to fluctuations in temperature, seawater salinity, and quantity/quality of light, leading them to adapt and develop protective strategies, such as increasing the content of some pigments to deal with light-irradiance fluctuation [5]. *Sargassum* genus, which comprises more than 350 species [6], is mostly pelagic and is distributed worldwide, despite being found mostly in tropical and subtropical environments [7]. Lately, the two halopelagic species (*S. fluitans* and *S. natans*) of this genus have been responsible for *Sargassum* tides on the coast of the Caribbean and Western Africa, negatively impacting the marine ecosystems [7]. *Sargassum* species have been used for centuries in agriculture as fertilizers, although their applications may go beyond fertilization purposes. Indeed, they have shown particularly interesting properties for the development of plant-biostimulation strategies and/or abiotic-stress mitigation, such as drought [8,9]. Moreover, these seaweeds have been explored as raw materials for biofuel production (e.g., methanol and bioethanol), for pharmaceutical, cosmetic, and textile industries, for bioplastic development, and for bioremediation [10,11,12]. Recently, the species, *Polycladia myrica*, widespread throughout the Persian Gulf, northern Australia, Mediterranean Sea, Pacific Ocean, and Indian Ocean tropics and subtropics, was also found to contain phlorotannins with promising UVR-protective effects, granting them great interest for cosmetic applications [13].

Fucaceae is another large family of Fucales. This is a broadly distributed family in the intertidal areas of the Northern Hemisphere, with six taxa recognized: *Ascophyllum*, *Fucus*, *Hesperophycus*, *Pelvetia*, *Pelvetiopsis*, and *Silvetia*, of which *Ascophyllum*, *Fucus*, and *Silvetia* are the most prominent genera [14]. These genera have been traditionally used in human nutrition for decades and, more recently, explored as potential ingredients in functional foods due to their richness in bioactive compounds [15,16]. *Fucus* is vastly distributed across the regions covered by the Fucaceae family and comprises 10 accepted species, of which *F. vesiculosus* is the most well-known, whereas *Ascophyllum* is a monotypic genus represented by *Ascophyllum nodosum*. *F. vesiculosus* is a good source of iodine, an essential component of thyroid hormones, and therefore, has been used in traditional medicine for the treatment of thyroid disfunction, including goiter [17] and obesity [18]. In turn, *A. nodosum* is the principal seaweed used as a source of industrial- and commercial-plant biostimulants, since it is shown to effectively improve plant growth, mitigate some abiotic and biotic stresses, and simultaneously enhance plant defenses by the regulation of molecular, physiological, and biochemical processes [19,20]. Moreover, due to their richness in bioactive compounds, *Fucus* and *Ascophyllum* extracts have a high potential to be used as nutraceutics and in healthcare [21].

The remaining families of Fucales are less representative and account for less than ten genera: Himanthaliaceae, a monotypic family comprising only one the genus *Himanthalia* and one species (*H. elongata*), is native to the northeast Atlantic Ocean, but can also be found in the North Sea and the Baltic Sea; Durvillaeaceae, also monotypic (*Durvillaea*), is found in the Southern Hemisphere, where some of the species are used in traditional cuisine [22]; Notheiaceae, another monotypic family (*Notheia*) with only one known species (*N. anomala*) that is an obligate epiphyte commonly found on *Hormosira* species along the southern Australia coast [23]. In turn, *Hormosira*, a single genus that belongs to the monotypic family, Hormosiraceae, and is native to southeastern Australia and New Zealand, being among the most common intertidal seaweeds on rocky shores found in those regions [24]. Finally, Seirococcaceae is a family with five genera, with only six species occurring exclusively in the Southern Hemisphere, and with a relatively restricted distribution [25]. 

Fucales members, like brown algae in general, have the metabolic capacity to biosynthesize unique phenolic compounds, named phlorotannins (PTs). PTs have been under the spotlight of many investigations since these compounds can display numerous bioactive properties that give them great potential for application in distinct medical-industrial areas. Indeed, because of their antimicrobial and antioxidant properties, PTs find applications in the food packaging industry, particularly as preserving agents in meat wrapping films for improved shelf-life and meat sensorial properties [26]. In the cosmetic industry, phlorotannins are ingredients of growing interest owing to their anti-aging and UV-protecting actions [27]. In fact, these can already be found in the composition of some sunscreen products, such as lotions [28] and sticks [29].

## 2. Methods

The current review provides comprehensive information about the recent advances in phlorotannins research, particularly focusing on the Fucales order. All available information was collected via an electronic search of different scientific sources, including PubMed (https://www.pubmed.ncbi.nlm.nih.gov; accessed on 10 May 2022), ScienceDirect (https://www.sciencedirect.com; accessed on 10 May 2022), Google Scholar (https://www.googlescholar.com; accessed on 10 May 2022), Scientifc Electronic Library Online (SciELO) (https://www.scielo.org; accessed on 10 May 2022), World Intellectual Property Organization (WIPO) (https://www.wipo.int/; accessed on 6 June 2022), and European Patent Office (EPO) (https://www.epo.org/; accessed on 6 June 2022). The authors opted for the following keywords to find relevant studies: “phlorotannins” “Fucales” “Sargaceae” “Fucaceae”, “Durvillaeaceae”, “Himanthaleaceae”, “Hormosiraceae”, “Notheiaceae, “Seirococcaceae”, “Extraction”, “HPLC”, “Mass spectrometry”, and “NMR”. These terms were used alone or in combination using Boolean operators (“and”, “or”, “not”).

## 3. Phlorotannins and Main Classes in Fucale

**Figure 1:**
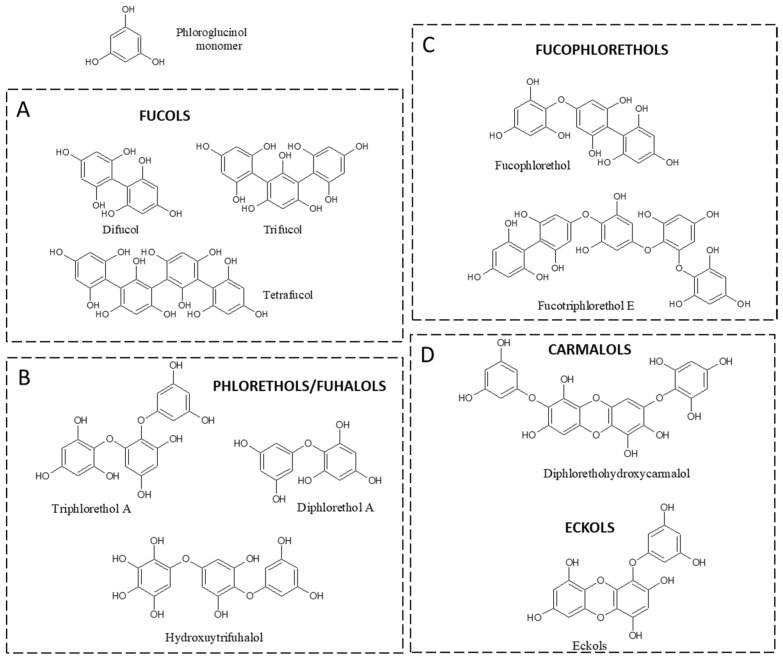
Classes of phlorotannins. (**A**) fucols containing only aryl bonds, (**B**) phlorethols and fuhalols containing only ether bonds, (**C**) fucophlorethols containing both aryl and ether bonds, and (**D**) carmalols and eckols containing dibenzodioxin bonds.

Chemically, PTs represent a class of dehydro-polymers of phloroglucinol (PHG) monomeric units (1,3,5-trihydroxybenzene), with a wide range of molecular weights (126 to 650 KDa), but, most commonly, the molecular weight of these biopolymers ranges from 10 to 100 kDa, divided according to the type of linkage among monomer units: fucols (-C-C- phenyl bonds), fuhalols and phloroethols (-C-O-C- ether bonds differed by their regular sequence of *para*- and *ortho*-ether bonds, by the presence of additional OH groups), fucophloroethols (both -C-C- and C-O-C- bonds), and eckols/carmalols (containing dibenzodioxin bonds). Eckols differ from carmalols by their usually low molecular weight and by the presence of a phenoxyl substitution (Figure 1).

Almost 90% of the total amount of PTs is found in a free state in physodes, that is, cell-cytoplasm, specialized membrane-bound vesicles, while the remaining are in the cell wall, complexed with alginic acid by covalent ester and hemiacetal bonds, acting as a structural component for osmotic-pressure regulation [30]. They are recognized to accumulate up to 25% of seaweeds’ dry weight (DW), although their levels are highly dependent on the algae species and other factors, including geographic region of growth, age, tissue type, water salinity, season, amount of nutrients, light intensity, and water temperature [31,32,33]. For example, among the seven species of Fucale, namely *Pelvetia canaliculata*, *Fucus spiralis*, *Fucus serratus*, *Bifurcaria bifurcata*, *Himanthalia elongata*, *A. nodosum*, and *F. vesiculosus*, the last two, known to develop mixed belts in the mid-tide zone, are shown to contain the highest content of phenolics (approximately 5.80% DW), while lower levels are found in species that grow in the lower-intertidal level (4.3% DW) and in the upper level of the intertidal zone (3.9% and 3.4% DW for *F. spiralis* and *P. canaliculata*, respectively) [34].

Lopes et al. [35] reported PT variations among species from two Fucale families, namely in *F. spiralis* (Fucaceae), *Cystoseira nodicaulis*, *Cystoseira tamariscifolia*, *Cystoseira usneoides*, and *Sargassum vulgare* (Sargassaceae), concluding that the maximal levels of PTs were found in the first one, representing about 12 times those found in *S. vulgare*. Intermediate and highly variable amounts were also detected among the three *Cystoseira* species.

The influence of geographical location and water salinity (positive correlation) on the content of PTs in *F. vesiculosus* from the Artic region was clearly shown by Obluchinskaya et al. [36], who registered levels between 72.4 and 158.1 mg PHG equivalents/g DW algae, depending on sampling locations. Moreover, Pedersen [37] reported that the phenolic content of *A. nodosum* and *F. vesiculosus* increased with increasing salinity in their habitats. Further research confirmed that the decrease in salinity matched with high exudation of *A. nodosum* and *F. vesiculosus* phenolics in the surrounding water, resulting in a significant reduction of the phenolic content of these two species [38].

Seasonal variation of PTs in Fucale has been screened in distinct species. When following the concentrations of PTs in five perennial-Sargassacean species from the coast of the Sea of Japan, Kamiya et al. [39] registered large variations throughout the year, but a relatively similar fluctuation pattern among the five species, consisting of a maximum in summer, followed by a decrease towards winter and an increase in April, was observed. In fact, although some contradictory results have been reported in the literature for Fucale species, such as *A. nodusum* and *Fucus*, most of the data suggest that the production of PTs is maximum during the summer, matching the period of the greatest sun-exposure period, and thus, agreeing with the UV-protective functions invoked for these compounds [34,40,41,42]. Interestingly, some authors have also reported higher production of PTs in seaweeds growing at higher latitudes, where water temperatures tend to be lower, rather than in lower latitudes, where water temperatures are more temperate. Indeed, the species, *Durvillaea antarctica*, from South-East Pacific, was found to contain considerably greater phlorotannin levels when collected in higher latitudes (closer to the South Pole) than those collected in mid and lower latitudes (closer to the equator) [43]. In part, the fact that this species is more adapted to subantarctic regions and strong wave forces, could explained such observations, since these can be an important factors for stimulating the synthesis of structural-function PTs rather than UV-protective ones, although this was not assessed by the authors.

Noteworthy, the aforementioned variables accumulate with changes in the degree of phlorotannin polymerization, which is also greatly influenced by algae species and biotic and abiotic restrictions, further increasing the structural diversity of PTs and making their elucidation difficult. In fact, most published data only reported total PTs levels among macroalgae samples, rather than elucidating differences in their profiles. In the case of Fucales, the analysis of the composition of PTs was restricted mainly to the families, Fucaceae and Sargassaceae, particularly, the genera *Fucus*, *Ascophyllum*, and *Cystoseira* [44,45,46,47]. 

In Fucaceae, *F. vesiculosus* is by far the most-studied species in relation to PTs. Among others, the UHPLC-DAD-ESI-MS^n^ analysis, carried out by our group on the ethyl acetate fraction of a hydroacetonic extract obtained from this algae species, revealed the presence of common fucols, fucophlorethols, fuhalols, together with several other PTs derivatives of varying degrees of polymerization, ranging from 3 to 22 phloroglucinol units. Moreover, possible new PTs, including fucofurodiphlorethol, fucofurotriphlorethol, and fucofuropentaphlorethol, have tentatively been identified in this species [48].

Notably, when analyzing PTs-enriched fractions of aqueous and hydroethanolic extracts of three macroalgae by UPLC-MS, Tierney and coworkers [45] suggested that *A. nodosum* and *P. canaliculata* contained predominantly larger PTs (degree of polymerization (DP) of 6–13 monomers), compared to *F. spiralis* (DP of 4–6 monomers). The complexity of PTs constituents was also referred to for Sargassaceae, particularly the *Sargassum* genus. In agreement with other work focusing on the *Sargassum* species, Li et al. [49] reported the predominance of fuhalol-type phlorotannins in a PTs-rich fraction of *S. fusiforme* (DP 2–10 monomers), and the detection of other relevant compounds, particularly phlorethols and fucophlorethols with varying degree of polymerization (DP 2–11 monomers); they also reported newly discovered eckols and carmalol derivatives. In general, the authors identify many challenges in the structural elucidation of PTs, which hinder the establishment of relationships between the composition of PTs and the bioactivity of the extract and, consequently, the full implementation of these natural resources by the industry. Thus, a concerted effort on the part of the algae community to develop effective and standard methods for the analysis of PTs is crucial.

## 4. Extraction Processes and Patents

Phlorotannins, as tannins in general, have been preferentially extracted using solid-liquid extraction (SLE) with hydroacetonic mixtures, although other solvents have been attempted. Concordantly, when comparing different extraction solvents, Wang et al. [50] obtained higher efficiency in extracting PTs from *F. vesiculousus* with hydroacetone (70%) rather than hydromethanol, hydroethanol, hydroethyl acetate, or water (at 20 °C or 70 °C), with a total of 393 mg PGE/g extract (78.6 mg PGE equivalents/g DW algae), although the highest extraction yield was obtained in water extracts. In turn, to optimize the extraction of PTs from the same species, Catarino et al. [48] recently tested various extraction conditions and concluded that extraction with 67% acetone (*v*/*v*), a solvent-solid ratio of 70 mL/g, and a temperature at 25 °C produced the highest extraction yield (28%). This is similar to the previous study, but with a lower content of total phlorotannin (10.7 mg PGE/g extract), highlighting the variability among algae samples. Recently, natural deep eutectic solvents (NADESs) were developed, consisting of mixtures, with different proportions of choline chloride, lactic acid, malic acid, betaine, glucose, and glycerin [51]. Although it was an SLE extraction, it can be considered an evolution towards greener methods due to the solvent used. Moreover, the authors found that using water solutions of the prepared NADES can improve the extraction yield of PTs from the algae, *F. vesiculosus* and *A. nodosum*, achieving values similar to those obtained with the most common organic solvents [51].

More efficient and environmentally friendly extraction procedures, including microwave-assisted extraction (MAE), ultrasonic-assisted extraction (UAE), pressurized solvent extraction (PSE), supercritical fluid extraction (SFE), and enzyme assisted extraction (EAE) have recently emerged as alternative methods to the conventional SLE extractions using organic solvents. All these new techniques were already applied to extract compounds from algae or, at least, to obtain algal extracts enriched in some compounds [52,53,54]. So, their application to PTs extraction was expected. In fact, recently, Meng et al. [55] reviewed these techniques’ application to PTs extraction from several brown macroalgae. In general, the included studies aimed to obtain rich extracts to evaluate their biological activities; therefore, the extraction methodology was not the main issue.

Nevertheless, it is interesting to notice that some groups changed their extraction methodology over the years and still obtained extracts rich in PTs. One of those examples is Valentão and co-workers who started using an SLE methodology with a mixture of acetone:water (7:3). Later on, the same group used the UAE technique to obtain PTs-rich extracts from several *Fucus* species. Although the authors’ aim was not to compare the two extraction methodologies and the amounts reported in the same cases but in different units, it seems that UAE did not improve the PTs amount in the studied macroalgae [16,56]. Another example that can be highlighted is Shikov and co-workers who used their previously prepared NADES to extract PTs from *F. vesiculosus* under UAE conditions. However, the extraction yield did not improve, compared to the authors’ previous data [51]. It is worth mentioning that the extraction time was reduced to 60 min, which is an advantage [57].

Focusing only on the extraction of PTs, it seems evident that SFE, although very efficient in the extraction of other metabolites, has not been extensively used in the extraction of PTs [55]. EAE seems to be an efficient methodology, but there are only a few reports [58], and its cost may be an obstacle to its large-scale use.

Few macroalgae species have been subjected to different techniques, making it difficult to establish a proper comparison because the amount of PTs extracted depends on the location and collection time [59,60]. For example, *F. vesiculosus* PTs were extracted using different percentages of ethanol and SLE [61], UAE [62], and PSE [63] techniques. The reported results showed that ethanol percentage influenced the PTs content, but it is also possible to conclude that PSE was the most efficient. A higher amount was extracted (3690 mg gallic acid equivalents/100 g DW seaweed) in just 4.68 min, whereas the other techniques used 30 min and 24 h, respectively, for UAE and SLE, and amounts below 60 mg of gallic acid equivalents/100 g DW seaweed. In addition, MAE was demonstrated to successfully extract PTs in larger amounts and with less extraction time [31,64,65]. This technique was also applied in *F. vesiculosus* [66], however, further studies are needed in Fucales species.

Although the use of non-conventional extraction methods is still scarce, a few considerations may be made regarding their use. MAE, UAE, and PSE require shorter extraction times, and, consequently, lower energy consumption. However, they may cause degradation if high temperatures are reached. They can be suitable for large-scale production, but the cost is relatively high. Naturally, SLE is the more accessible and less expensive technique to use on a large scale; unfortunately, it also involves solvents that are less environmentally friendly.

**Figure 2:**
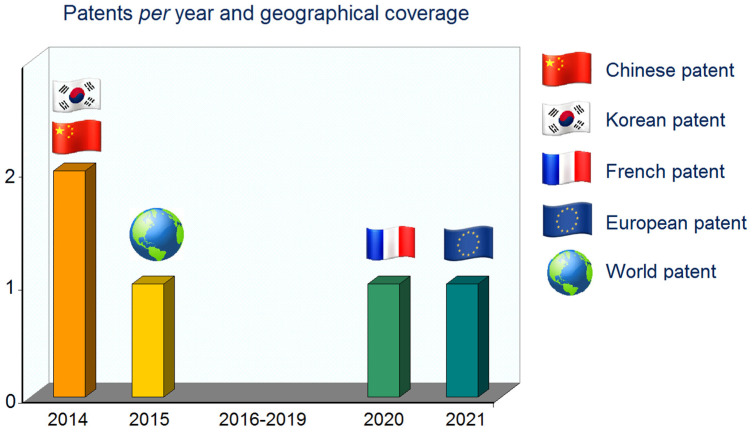
Distribution of the number of patents per year and its geographical coverage.

Table 1 summarizes distinct methods described in patent literature for the extraction of PTs from Fucales, and Figure 2 shows the distribution of the number of patents per year and geographical coverage. As Asian countries have a long tradition of using seaweeds and their derived products in food and cosmetics, it is not surprising to find patented processes for the extraction of PTs from seaweed registered in Asia. 

In tandem, some European and world patents are available, which demonstrates the growing interest in the applications of PTs. As for the extraction processes per se, it is worth noting that the use of SLE procedures is based on solvents that are safe to use in humans, such as water, glycerin, and hydroethanolic mixtures. The rationale behind the use of such solvents is linked to the intended uses of the extracts: claimed applications of PT-rich extracts include whitening, antioxidant, anti-inflammatory, and moisturizing agents in cosmetics [67,68,69,70], and functional ingredients in foods [71].

## 5. Methods for Characterization and Quantification

Despite the exponentially growing interest in PTs’ bioactivities and potential applications in a range of therapeutic strategies, one of the main struggles when dealing with these compounds is their chemical characterization and structural elucidation. Due to the huge chemical variability and complexity of PTs, allied to the limited availability of commercial standards, the analysis of PTs currently remains quite a challenging task. In fact, it is rather common to find work that does not go further beyond the estimation of PTs using colorimetric methods [56,72,73]. Nevertheless, much effort has been taken towards the elucidation of the structural features of PTs, which have already provided relevant insights into the chemistry of these compounds.

In this field, liquid chromatography combined with mass spectrometry continues to stand as a powerful analytical tool for rapid analysis of these compounds, although there are some nuances when comparing with land phenolics. This topic advances the discoveries made over the past years regarding methods used for separating and identifying PTs from Fucales.

### 5.1. Spectrophotometric Quantification of Phlorotannins

Spectrophotometric methods for the quantification of PTs offer a rapid and simple approach to assessing PTs content in a given sample or extract. As phenolic compounds, Folin-Denis or Folin-Ciocalteu (FC) methods remain viable approaches for monitoring total PTs content. Both methods are based on the well-known chemical-phenolic-mediated reduction of a phosphotungstic-phosphomolybdic reagent (FC reagent) forming a blue color with an absorption spectrum at approximately 760 nm [74]. However, even though PTs are the major phenolic compounds present in brown seaweeds, several other non-phlorotannin phenolic compounds are also present [75]. Moreover, this reagent lacks sensitivity since it does not react only with phenolics, but also with any reducing substance (e.g., sugars, proteins, vitamins, thiols, and inorganic ions) in the sample, thereby, measuring its total reducing capacity [76]. Therefore, when the focus is the PTs, this may not be the most suitable approach as these unspecific reactions may lead to false reading and erroneous estimations. A more specific method has been developed by Stern et al. [77] using 2,4-dimethoxybenzaldehyde (DMBA) to react specifically to 1,3- and 1,3,5-substituted phenols, such as phloroglucinol and PTs, and form triphenylmethane pigments that can be monitored in the range of 510 nm. However, this reaction depends on the compounds’ branching degree or the presence of additional hydroxyl groups, such as fuhalols. Because the reaction with DMBA must occur between the hydroxyl groups through the two, four, or six positions, substituting these positions will hinder chromophore formation, eventually resulting in underestimations.

Although these methods are considered useful for a quick and preliminary estimation of PTs content of numerous samples, their major drawback is their incapacity to accurately quantify and identify individual compounds, which makes it necessary to turn to more sophisticated and powerful separative and analytical techniques to enable proper elucidation of the structural features of PTs.

### 5.2. Methods for Phlorotannins’ Separation and Identification

#### 5.2.1. HPLC-DAD

Because of their structural similarity and complexity, PTs analysis and identification of individual compounds remain quite a challenging task. One of the major problems faced in PTs analysis is their chromatographic separation. The close similarities of the compounds structures and the possibility of forming multiple isomers make it difficult to achieve a proper separation of individual PTs when using HPLC, and even in its upgraded variant, UHPLC. In this field, the literature is divided into two main approaches: reverse- and normal-phase chromatography. On one side, although reverse chromatography is useful for separating compounds with a similar structure within complex samples, the high polar nature of PTs gives them a particular affinity with the mobile phase, resulting in very fast elutions and forming the typical, poorly resolved “humps” [78]. This technique has, however, been widely used for the analysis of PTs of multiple Fucales, including several species from the genus *Fucus* (*F. vesiculosus*, *F. guiryi*, *F. serratus*, and *F. spiralis*), *Cystoseira* (*C. nodicaulis*, *C. tamariscifolia*, and *C. usneoides*), *A. nodosum*, and *B. bifurcata*, among others; and although it usually does not allow to separate compounds longer than 8–10 units, it has been very useful for accomplishing valuable insights regarding the low polymerization degree of PTs’ profile of these seaweeds [16,47,48,50,79]. On the other side, normal phase involves the use of a polar-stationary phase that better retains these high-polar compounds. The use of this technique for PTs separation and isolation was more frequent in the past, with authors applying pre-acetylation of the compounds. Indeed, using this technique Glombitza and his co-workers were able to isolate and characterize several PTs from the genera *Cystophora*, *Sargassum*, and *Carpophyllum* [80,81,82,83], and even identify halogenated PTs in *Carpophyllum angustifolium* [84] and in different species from the genus, *Cystophora* [85,86]. More recently, Koivikko et al. [87] developed a NP-HPLC method suitable for separating *F. vesiculosus* PTs according to their degree of polymerization, without requiring a previous acetylation step, and capable of producing a chromatogram with improved retention times and peak definition, compared with RP-HPLC. This technique has also been used for the analysis of PT metabolites in urine samples of *A. nodosum*-administered human volunteers [88]. The major issue of NP-HPLC is that higher PTs with higher number of hydroxy groups establish strong interactions with polar stationary phases, which make them very difficult to elute. Moreover, interfacing typical NP solvents with ESI can be challenging due to reduced sensitivity caused by poor ionization efficiency in 100% non-polar solvents [78].

**Figure 3:**
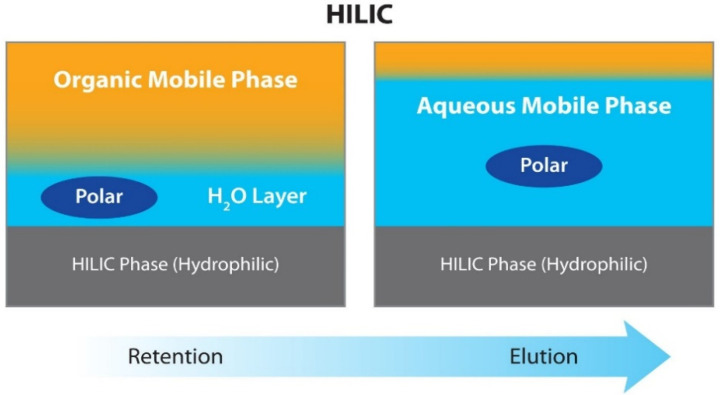
Schematic representation of the interactions between stationary and mobile phases in HILIC columns. Adapted from https://www.restek.com/row/technical-literature-library/articles/how-to-avoid-common-problems-with-hilic-methods/ (accessed on 28 November 2022).

Alternative methods have been recently explored to overcome these issues. This is the case of hydrophilic interaction liquid chromatography (HILIC), considered as a variation of the normal-phase liquid chromatography, and is characterized by the combination of hydrophilic stationary phases with reverse phase-low aqueous eluents, promoting the formation of two layers in the mobile phase, that is, a water-rich layer forms on the surface of the polar-stationary phase and another organic-rich layer forms on top of that (Figure 3), creating a liquid/liquid extraction-like system inside the column [89]. In this chromatographic mode, the analytes are distributed between these two layers and eluted according to the partitioning equilibrium shifts occurring between these two phases, although other interactions, such as dipole-dipole, hydrogen bonding, and electrostatic forces also play an important role [90]. 

These characteristics make HILIC columns the most adequate for separating PTs, allowing them to establish strong interactions with the polar-stationary phase, consequently increasing their retention times and separation. This technique has been applied for separating PTs from extracts of different Fucales, namely *F. vesiculosus*, *F. spiralis*, *A. nodosum*, *S. longicruris*, and *P. canaliculata*, allowing to separate and detect compounds with polymerization degrees that ranged from 3 to 49 units [78], which is far beyond the reach reported by Tierney et al. [45], who performed a similar study using RP column for profiling *F. spiralis*, *A. nodosum*, and *P. canaliculata*, and only managed to achieve the detection of compounds with DPs of up to 16 units [45].

**Figure 4:**
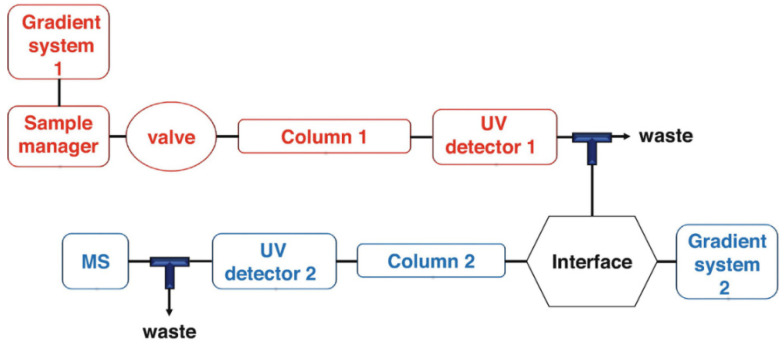
Schematic representation of a 2D LC chromatographic system.

More recently, 2D LC techniques (Figure 4) have been successfully employed to assess complex mixtures of PTs in seaweed extracts, achieving significantly improved separations. In this approach, samples are injected in an apparatus containing two separating columns connected sequentially so that the eluent that exits from the first column is transferred to the second column, which runs using different conditions. Typically, the second column has a separating mechanism that complements the first column, so that the poorly resolved peaks from the first column can be properly separated in the second [91]. In the particular case of PTs, the most frequently reported set ups describe the combination of HILIC columns (first dimension) for separating the compounds according to their polymerization degrees, with RP columns (second dimension), which allows for separation according to their hydrophobicity. Using this technique, Montero et al. were able to separate and detect over 50 compounds in the species, *Cystoseria abies-marina* [4], and more than 70 compounds in *Sargassum muticum* [92]. Table 2 compiles the different chromatographic configurations and conditions that have been used through time for the separation and detection of PTs in Fucales.

Naturally, regardless of the columns used, U/HPLC always requires coupling to a detection system to monitor the compounds as they elute. UV-Vis detectors, especially photodiode arrays (PDAs), are arguably the most common detectors hyphenated with HPLC since many natural compounds of interest absorb in the UV-Vis region (from 190–600 nm) [93]. Phenolics, in particular, usually exhibit strong absorbances around 270–280 nm, and PTs are no exception, as their absorbance maxima is close to 270 nm [94]. The identification of compounds using HPLC-PDA can be achieved by comparing the UV spectrum and retention time of a given compound with that of its commercial standard. Moreover, this technique is very useful for determining the content of individual compounds in complex samples, as the concentration of such compounds, outputted as absorbance, can be quantitatively measured using the Beer’s Law [95]. However, the application of this approach to identify and quantify PTs is very limited since there are only a small number of commercial standards available for this purpose. Therefore, the identification of PTs usually requires more powerful techniques, such as mass spectrometry (MS) or nuclear magnetic resonance (NMR).

#### 5.2.2. Mass Spectrometry

**Figure 5:**
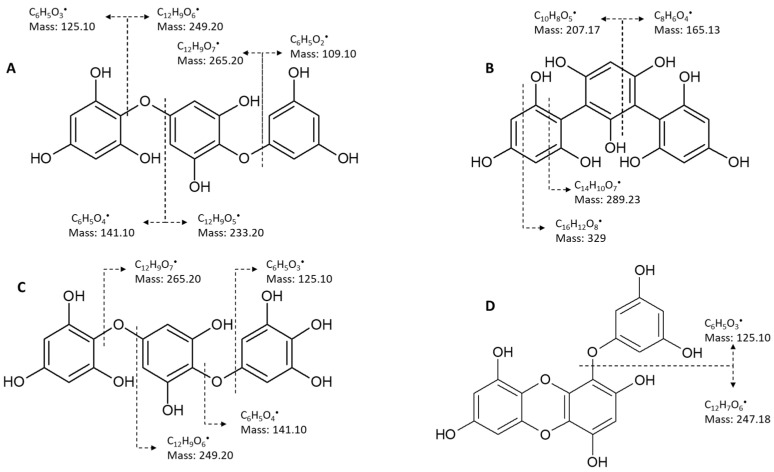
Examples of some fragment patterns of the different phlorotannin types, including phlorethols (**A**), fucols (**B**), fuhalols (**C**), and eckols (**D**), observed by mass spectrometry.

The use of MS directly coupled to U/HPLC instruments has revolutionized the characterization of complex extracts, providing high sensitivity and improving analytic capacity in a relatively short period of time. For that reason, this technique has become a stable practice for the characterization and identification of phenolic compounds in general, including PTs. Tandem MS or MS/MS allows the detection of mass-to-charge ratio (*m/z*) of the compounds’ pseudo-molecular ions (usually generated via electrospray ionization) as they elute from the column, and further obtains their respective fragmentation patterns via collision-induced dissociation (CID) [118]. This is a crucial step in the assignment of PTs because the formed fragments can provide important information about the structural features of the parent ion that originated them. Typically, almost every PT presents a base peak at MS^2^ that corresponds to the loss of one or two water moieties [46]. Likewise, the loss of 126 Da is a common feature of these compounds since this corresponds to phloroglucinol moiety, which is the building block of PTs [119]. The appearance of certain neutral lossesto the detriment of others is important to understand the type of linkages established between phloroglucinol units. For example, assuming that C−C bonds usually require higher energy to break than C−O−C linkages, fucols, phlorethols, and fucophlorethols with the same degree of polymerization will display the same molecular weight, but eventually produce different fragmentation patterns. In phlorethols, fragmentation can occur in either site of the ether bond, generating neutral losses of 126, 142, or 110 Da (Figure 5A), corresponding to phloroglucinol, tetrahydroxy benzene, or resorcinol, as well as their combination with additional phloroglucinol or water units. Contrastingly, the breaking of C-C bonds characteristic in fucols can only give rise to neutral losses of 126 Da (phloroglucinol), and because these bonds require higher energy to break, fucols also frequently present neutral losses of 14, 28, 44, and 84 Da (Figure 5B), as well as their combinations with additional phloroglucinol or water moieties, which are indicative of cross-ring cleavages. Compounds that display both types of cleavages are most likely to be fucophlorethols (Figure 5A,B) [48]. Fuhalols, on the other hand, are slightly easier to distinguish since these compounds have a phlorethol-like arrangement that contains at least one additional hydroxyl group. Therefore, the pseudo-molecular ion of a fuhalol with a given polymerization degree always presents a *m*/*z* 16 Da higher, compared with its phlorethol equivalent (Figure 5C). Moreover, the MS/MS spectra of these compounds frequently present multiple product ions and/or neutral losses corresponding to tetrahydroxy benzene and its combination with phloroglucinol (e.g., [M-H]^−^ at *m*/*z* 265, 389, 513, and so on) [92].

As for eckols, due to the presence of dibenzodioxin structures, these can be distinguished from the other groups based on their deprotonated molecular ions, which are usually 2 Da lower than other PTs with equal DPs, for each dibenzodioxin motif present in the structure (Figure 5D). For instance, eckol presents a [M-H]^−^ at *m*/*z* 371, while trifucol, triphlorethol, and fucophlorethol are all present [M-H]^−^ at *m*/*z* 373. In turn, bieckols, which have two dibenzodioxin structures in its backbone, present a [M-H]^−^ at *m*/*z* 741, while their hexamer equivalents present [M-H]^−^ at *m*/*z* 745. The same applies to carmalols, which are essentially fuhalols containing dibenzodioxin structures [120]. Finally, some PTs that contain furan rings, such as fucofuroeckol and phlorofucofuroeckol, are characterized by a dehydrated-deprotonated molecular ion, i.e., a [M-H]^−^ that is 18 Da lower, compared to their non-furan-containing equivalents with equal DPs (e.g., fucofuroeckol vs. phloroeckol structures only differ on the presence/absence of the furan ring and show [M-H]^−^ at *m*/*z* 496 and 478, respectively) [120]. Table 3 shows MS data collected from different studies focused on the identification of PTs in Fucales.

Although these fundaments may be useful to achieve reasonable structural identification of low molecular-weight PTs, major difficulties arise when it comes to oligomers and polymers of high molecular weights since their isomerization increases exponentially, and the multiple possibilities for phloroglucinol arrangements and combinations can hardly be elucidated by MS or MS/MS. Nevertheless, in such cases, MS can still provide valuable information about the DP of the compounds present in a phlorotannin mixture. In this regard, matrix-assisted laser desorption/ionization time of flight MS (MALDI-TOF-MS) constitutes a more suitable approach for the analysis of larger oligomers since it can detect molecules with *m/z* above the upper limit of ESI-MS. This technique has been used to study the phlorotannin fraction of *Sargassum wightii*, allowing to confirm the presence of dimers, trimers, and hexamers of phloroglucinol [125]. High-resolution MS (HRMS) is an alternative approach that can also be used for the analysis of PTs with high molecular weights, relying on the multiple-charged ions, and changes in the +1 ^13^C isotope pattern. In other words, when a compound is double- or triple-charged, it shows a +1 ^13^C isotope pattern with *m/z* differences of 0.5 and 0.33, respectively. On this principle, Steevensz et al. [78] were able to profile the PT composition of *P. canaliculata*, *F. spiralis*, *F. vesiculosus*, and *A. nodosum* in terms of DP, detecting compounds with molecular weights up to 6000 Da that, otherwise, would exceed the mass range of ESI-MS.

Overall, although mass spectrometry can be a resourceful technique for the qualitative analysis of PTs and even powerful enough to achieve reasonably good characterization of low molecular-weight compounds, it fails to retrieve sufficient details on the type and linkage position of the isomeric forms of compounds with higher DPs. Therefore, when the goal is to fully elucidate PTs-structural features, NMR is the only effective method that can offer a sufficient resolution for such an in-depth analysis.

#### 5.2.3. NMR

NMR spectroscopy comprises a direct and non-destructive analysis that can be useful not only to access the contents of PTs, but also to fully elucidate the structural features of PTs. When quantitative data is intended, ^1^H NMR resonance signals of all phenolic compounds contained in algal extracts are integrated and compared with those of an appropriate internal standard; these compounds need to be stable, chemically inert, available in highly pure form, and completely soluble in the same deuterated solvent(s) as the sample. Overall, authors have been using different approaches and methodologies to quantify phenolic compounds and/or PTs through this technique, including in Fucale-derived samples.

In 2009, Parys et al. described, for the first time, the use of quantitative ^1^H NMR (qHNMR) to determine the variability of phlorotannin content of *A. nodosum* during the course of the year. In their work, trimesic acid was used as the internal standard (2.0 mg oftrimesic acid in 0.8 mL of deuterated methanol and 0.2 mL of deuterium oxide), and calibration curve was produced using phloroglucinol. Despite higher PTs content being detected by qHNMR, compared to those obtained using the FC colorimetric method, both methods followed the same seasonal-variations trend [126]. It is important to note that, in contrast with colorimetric assay, which is not specific for PTs or even for phenolic compounds, the integration of the resonance signals of ^1^H NMR spectra in qHNMR is compared with those of the internal standard. Due to these principal differences, a direct comparison cannot be accurately made between both methodologies [127,128].

Using a distinct approach, Stiger-Pouvreau and co-workers used high-resolution magic angle spinning (HR-MAS) to quantify PTs in the brown algae, *Cystoseira tamariscifolia*. In this study, solid-state NMR was used to detect phloroglucinol (monomer) in vivo and ^1^H qNMR was applied to estimate the monomer quantification (using trimethylsilyl- propionate –d4 (TSP) as an internal standard). The phloroglucinol singlet at *δ* 6.02 ppm, integrated into three protons, confirmed its presence within the sample. The accuracy of this method was assessed using a standard solution of phloroglucinol and the results were validated by comparison with those of FC assay. Ultimately, the authors claimed that the presented methodology consists of an innovative and rapid method to quantify phloroglucinol, which can be applied to all algal species. The only limitation of qNMR method lies in the necessity of at least one of the spectra signals (singlet, doublet, etc.) being unambiguously attributed to one and only one compound [129].

Earlier in 2010, the same authors already demonstrated the potential of in-vivo ^1^H HR-MAS NMR associated with Mass Spectrometry (LC/ESI–MS^n^) to observe the global chemical profile of five species of the genus, *Cystoseira*, present along the coasts of Brittany in France: *C. baccata*, *C. foeniculacea*, *C. humilis*, *C. nodicaulis*, and *C. tamariscifolia* [130]. The main objective of their work was to identify *Cystoseira* specimens and discuss their taxonomy. The results proved the efficiency of the presented approach to distinguish mainly *C. nodicaulis* and *C. tamariscifolia* as their spectra evidenced the presence of characteristic signals, allowing their unambiguous identification. In the case a singlet at 2.91 ppm for the first and for the latter, a peak at precisely 6.00 ppm characterized the species and indicated the possible occurrence of a simple phlorotannin. *Foeniculacea* and *C. humilis* were generally characterized by the occurrence of two doublets of equal intensity at 7.90 and 7.36 ppm. However, the absolute discrimination between the two signals remained impossible. The similarity of the in-vivo NMR signals, in tandem with the slight intraspecific chemical diversity of both species, justified these results. Lastly, in the case of *C. baccata,* despite showing the most important chemical diversity, many signals permitted constant discrimination from the other species.

In 2020, Walsh and co-workers [131] demonstrated the antimicrobial potential of two purified phlorotannin extracts from *A. nodosum* and *F. serratus*, two intertidal brown seaweeds. In that work, ^1^H and ^13^C NMR analysis allowed not only a quantitative and qualitative estimation of total phenolic compounds, but also access to differences in the linkage profile between purified-phenolic extracts of both species. As for FC assay, a significantly higher level of total-phenolic compounds was found in *A. nodosum* than in *F. serratus*, and the results were validated by FC assay. Moreover, the achieved results using both qNMR and FC assay clearly demonstrated the existence of variations between samples collected each month and between both methodologies.

To elucidate the PTs structure, ^1^H and ^13^C NMR analysis were first applied by Glombitza and his group in the ‘70s, when they identified phloroglucinol in different brown algae species. The complexity of spectra associated with these derivatives only allowed for the identification of smaller polyphenolic structures. Thus, in 1974, it was the first time when the chemical structures of bifuhalol and diphlorethol were elucidated from an 80% ethanol extract of *C. tamariscifolia* [132].

**Figure 6:**
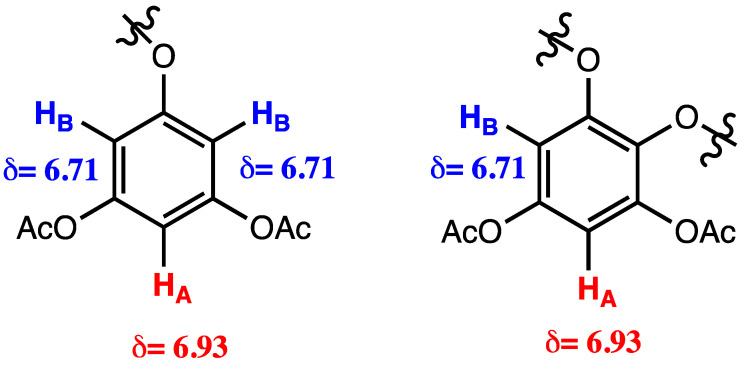
Types of aromatic-rings systems used to describe phlorotannins structures.

Nevertheless, since then, more than one hundred PTs structures have been elucidated using NMR [133]. For that, extracts are usually submitted to a pre-treatment step with hexane or petroleum ether to precipitate large PTs. Additionally, to prevent PTs instability, facilitate NMR analysis, and change the polarity of these compounds, they are often acetylated using acetic anhydride and pyridine, thereby, allowing their normal-phase-silica-chromatography purification [119]. In the ^1^H NMR spectra of this type of compounds, two aspects must be remarked: the resonance of aromatic protons appears between *δ* 6.0 and 7.5 ppm, and the acetyl groups appear as singlet peaks between *δ* 2 and 3 ppm. This is a useful tool to establish the number of free-hydroxy groups in the original unprotected phenolics. The two types of aromatic-ring systems that may occur are represented in Figure 6. The distinctive chemical ambient of the two types of protons (Ha and Hb) alter the multiplicity of the observed signals in ^1^H NMR, as well as their integration. 

Together with ^1^H NMR spectroscopy, ^13^C and HSQC, and HMBC NMR spectra, have been used to clarify the structures of purified PTs, including in Fucales. The heteronuclear-correlation-NMR-spectroscopic approach is useful to identify the class of PTs from a sample matrix (monomer, fucol, phloroethol, fulahol, fucophloroethol, etc.) [134]. The characteristic ^13^C-NMR signals of PTs are summarized in Table 4. Appearing at characteristic-chemical shifts, carbon resonances of a distinct type of carbons together with their intensity allow the attribution of some signals to specific classes of PTs, particularly the phlorethols e fuhalols.

**Figure 7:**
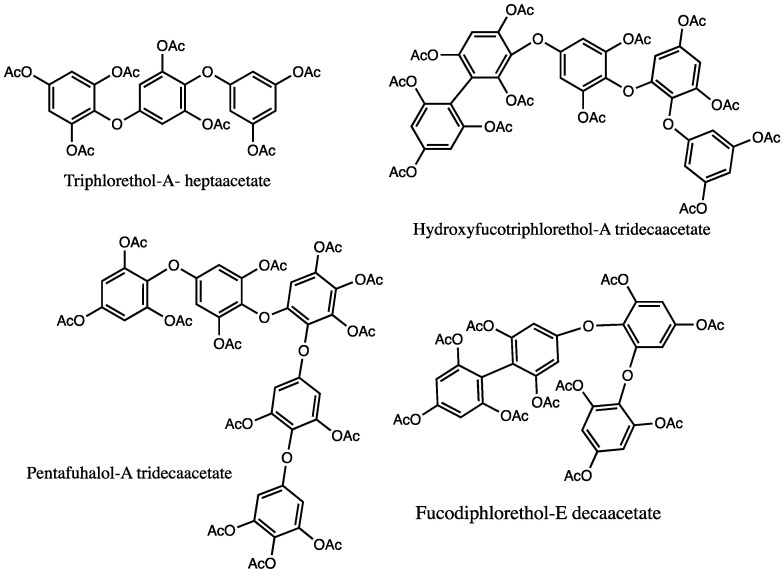
Examples of phlorotannins isolated and characterized (using NMR) from *Cystophora torulosa*.

In 1997 Glombitza et al. [135] described the isolation and characterization of 33 PTs from the brown alga, *Cystophora torulosa*. Different PT classes were identified including phlorethols and fuhalols, and fucophlorethols and hydroxyfucophlorethols (examples in Figure 7). Moreover, regarding the latter, seven new hydroxyfucophlorethols bearing additional hydroxyl groups were identified and characterized (NMR and MS). As previously referred, to prevent oxidation and increase the lipophilicity of the isolated PT derivatives, the authors described their isolation as acetates [82].

Koch et al. have also demonstrated the application of ^1^H and ^13^C NMR to characterize larger fuhalolacetates in *B. bifurcata* [136]. HSQC and HMBC (2D) NMR spectroscopic techniques have been used by Cérantola et al. to display the presence of fucol and fucophlorethol structures in the extracts of *Fucus spiralis* [137]. The same approach was used with *Halidrys siliquosa*, abundant in Brittany. The use of 1D and 2D NMR, and MS analysis permitted the identification of four phenolic derivatives: trifuhalols and tetrafuhalols and, for the first time, diphlorethols and triphlorethols [125]. Table 5 summarizes the studies mentioned above in which ^1^H and ^13^C NMR contributed to the elucidation of the structure of PTs extracted from algae belonging to Fucales.

## 6. Concluding Remarks

Summing up, Fucales comprise a vast group of seaweed species with a great variability in terms of phlorotannin compounds. Spectrophotometric assays can be a useful tool for high-throughput, easy, and cost-effective screening of phlorotannin contents. However, to separate, quantify, and characterize these compounds, robust analytical techniques are essential. Currently, MS coupled to HPLC offers a satisfactory approach for the separation and characterization of oligomeric phlorotannins. Significant improvements were also brought with the development of more specialized equipment, such as UHPLC and HRMS. However, when full details on the linkage positions and isomeric forms are necessary, only NMR can offer that capacity. Nevertheless, this equipment is not the most affordable/accessible for laboratories. The availability of more standard compounds could contribute to a better use of HPLC as it would generate reliable libraries for comparison. Alternatively, to study and identify the common PT compounds by NMR spectroscopy, linking them to HPLC retention times and UV-spectral data could represent another step forward for the research community.

## Figures and Tables

**Table 1:** Patented methods for preparing phlorotannin-containing extracts from brown algae.

Raw Material	Preparation of the Crude Extract		Secondary Treatment of the Crude Extract	Phlorotannin Content	Ref.
	Solvent	Conditions			
*Bifurcaria bifurcata* dry powder	5% algae in mixture of water and glycerin (60:40)	RT time = 30 min pH = 8	setting pH at 4–5, filtering (nylon, 0.2 μm mesh)	0.23 ± 0.03 g/L	[69]
*Sargassum fusiforme* dry powder	water (96% wt)	temp = 55 °C time = 48 h enzyme is added	filtering and extracting solid residue with 40 L of ethanol (50% vol) for 12 h; concentrating both filtrate and secondary extract (6 h vacuum), spray-drying them and mixing them to obtain solid extract	—	[71]
*Fucus vesiculosus* dry powder	water (95% wt)	pre-soak (1 h) temp = 50–60 °C time = 120 min stirring	centrifuging and extracting solid pellet with 1 L of water (90 min, 50–60 °C); merging two liquid extracts, adding activated carbon (0.5%), stirring (15 min, 100 °C), filtering	—	[67]
*Fucus spirallis* dry powder	ethanol/water (70%)	soxhlet extraction followed by drying in rotating evaporator at 40 °C	re-suspending in water at 80 °C, filtering (paper), extracting liquid with ethyl ether and ethyl acetate	—	[68]
*Ascophyllum nodosum* dry powder	mixture of water and ethanol (pref. 75:25)	accelerated solvent extraction in a 10 mL-chamber, added with Fonteinebleau sand temp = 150 °C time = 2 × 5 min pressure = 10^6^ Pa	drying liquid extract using rotating evaporator and freeze-drier sequentially	150 g/kg	[70]
*Halidrys siliquosa* dry powder	mixture of water and ethanol (pref. 75:25)	accelerated solvent extraction in a 10 mL-chamber, added with Fonteinebleau sand temp = 150 °C time = 2 × 5 min pressure = 10^6^ Pa	drying liquid extract using, sequentially, rotating evaporator and freeze-drier	119 g/kg	[70]

**Table 2:** Chromatographic conditions used for separation and identification of phlorotannins in Fucales.

Species [Ref]	Configuration	Column	Mobile Phase	Gradient
*Durvillaea antarctica* [95]	RP-HPLC-DAD-ESI-MS/MS (+/−)	Unspecified C18 (5 μm, 4.6 mm ID × 25 cm)	A: 1% FA; B: ACN; flow rate: 1 mL/min	%B (min): 5 (0–5); 5–30 (60); 30–60 (70); 60 (80)
*Ascophyllum nodosum* [44]	RP-UHPLC-DAD-ESI-MS/MS (−)	CSH Phenyl-hexyl (2.1 mm ID × 100 mm, 1.7 µm)	A: H_2_O; B: ACN 95%; flow rate: 0.3 mL/min	%B (min): 5 (0–15); 5–30 (30); 30–100 (35); 100/47)
*A. nodosum* [88]	RP-UHPLC-DAD-ESI-MS/MS (−)	Zorbax SB C18 (2.1 mm ID × 100 mm, 1.8 μm)	A: 0.1% FA; B: 0.1% FA in ACN; flow rate: 0.2 mL/min	%B (min): 10 (0–3); 40 (15); 70 (40); 70 (50)
*Pelvetia canaliculata*, *Fucus spiralis*, *F. vesiculosus*, *A. nodosum* [78]	HILIC-UHPLC-DAD-ESI-HRMS (−)	BEH Amide (2.1 ID × 100 mm, 1.7 μm)	A: 10 mM ammonium acetate; B: ACN; flow rate: 0.4 mL/min	%A (min): 5 (0–1); 35 (17)
*A. nodosum*, *P. canaliculata*, *F. spiralis* [45]	RP-UHPLC-DAD-ESI-MS (−)	HSS PFP (2.1 ID × 100 mm, 1.8 μm)	A: 0.1% FA; B: 0.1% FA in ACN; flow rate: 0.5 mL/min	%B (min): 0.5 (0–10); 30 (26); 90 (28)
*F. vesiculosus* [46,48,66,96]	RP-UHPLC-DAD-ESI-MS (−)	Hypersil Gold C18 (2.1 ID × 100 mm, 1.9 μm)	A: 0.1% FA; B: ACN; flow rate: 0.2 mL/min	%A (min): 5 (0); 40 (14.7); 100 (16.6); 100 (18.8)
*A. nodosum* [97]	RP-HPLC-DAD	Sun Fire C18 (4.6 ID × 250 mm, 5 μm)	A: 0.05% orthophosphoric acid; B: ACN; flow rate: 0.8 mL/min	%B (min): 0 (0–4); 30 (8); 60 (16); 70 (20)
*F. serratus*, *F. vesiculosus*, *Cystoseira nodicaulis*, *H. elongata* [98]	RP-UHPLC-DAD-ESI-MS (−)	HSS PFP (2.1 ID × 100 mm, 1.8 μm)	A: 0.1% FA; B: 0.1% FA in ACN; flow rate: 0.5 mL/min	%B (min): 0.5 (0–10); 30 (26); 90 (28)
*F. vesiculosus* [99] P-UHPLC-DAD-ESI-MS/MS (−)	HSS PFP (2.1 ID x 100 mm, 1.8 μm)	A: 0.1% FA; B: 0.1% FA in ACN; flow rate: 0.5 mL/min	%B (min): 0.5 (0–10); 30 (26); 90 (28)	
*Carpophyllum flexuosum* [100]	RP-HPLC-DAD-ESI-MS (+)	Phenomenex C18 (4.6 ID × 250 mm, 5 μm)	A: 0.1% FA; B: ACN; flow rate: 0.8 mL/min	%B (min): 0 (0); 60 (60)
*F. vesiculosus* [50]	RP-HPLC-DAD-ESI-MS/MS (−)	Zorbax SB-C18 (4.6 mm ID × 250 mm, 5 μm)	A: 0.1% FA; B: 0.1% FA in ACN; flow rate: 1 mL/min	%B (min): 10 (0–5); 26 (15); 30 (30); 44 (32); 60 (42)
*A. nodosum*, *B. bifurcata*, *F. vesiculosus* [79]	RP-HPLC-DAD–ESI-MS/MS (−)	Zorbax SB-C18 (3.0 mm ID × 150 mm, 3.5 μm)	A: 2.5% HOAc; B: 2.5% HOAc in MeOH; flow rate: 1 mL/min	%B (min): 5 (0); 15 (15); 30 (35); 40 (40); 60 (50); 90 (55); 100 (55.01), 100 (75)
*A. nodosum* [101]	RP-HPLC-DAD-ESI-MS/MS (−)	Synergi Hydro C18 (2.0 ID × 150 mm, 4 μm)	A: 0.1% FA; B: 0.1% FA in ACN; flow rate: 0.3 mL/min	%B (min): 2 (0–2); 5 (5); 45 (25); 100 (26); 100 (29)
*F. vesiculosus* [87,102]	NP-HPLC-ESI-MS (−)	LiChrospher Si 60 (4.0 mm ID × 250 mm, 4 μm)	A: 82:14:2:2 DCM:MeOH:H_2_O:HOAc; B: 96:2:2 MeOH:H_2_O:HOAc; flow rate: 1 mL/min	%A (min): 100 (0–30); 17.6 (45); 30.7 (50); 87.8 (60)
*P. canaliculata*, *F. vesiculosus*, *A. Nodosum*, *H. elongata* [103]	RP-UHPLC-DAD-ESI-MS (−)	HSS PFP (2.1 ID × 100 mm, 1.8 μm)	A: 0.1% FA; B: 0.1% FA in ACN; flow rate: 0.5 mL/min	%B (min): 0.5 (0–10); 30 (26); 90 (28)
*F. vesiculosus* [104]	RP-HPLC-DAD-ESI-MS (−)	Nucleodur 100 (2 mm ID × 100 mm, 5 μm)	A: 2mM NH_4_Ac; B: 2mM NH_4_Ac in MeOH	%B (min): 10 (0); 100 (20)
*F. vesiculosus* [16]	RP-HPLC-DAD-ESI-MS/MS (−)	Kinetex (4.6 mm ID × 150 mm, 5 μm)	A: 0.1% FA; B: ACN; flow rate: 0.8 mL/min	%B (min): 1 (0); 10 (12); 30 (25); 50 (27); 50 (28)
*F. vesiculosus* [105]	RP-UHPLC-DAD-ESI-MS/MS (+/−)	Phenomenex Prodigy (2 mm ID × 150 mm, 3 μm)	A: 20 mM FA, 60 mM NH_4_HCOO/FA in water; B: ACN; flow rate: 0.3 mL/min	%B (min): 2 (0–2); 40 (16); 100 (18)
*F. spiralis*, *C. usneoides*, *C. tamariscifolia*, *C. nodicaulis* [47]	RP-HPLC-DAD-ESI-MS/MS (+)	Luna C18 (4.6 mm ID × 250 mm, 5 μm)	A: 0.1% FA; B: ACN; flow rate: 1 mL/min	%B (min): 0 (0–10); 30 (30); 80 (35); 80 (40)
*F. vesiculosus* [106]	RP-HPLC-DAD-ESI-MS/MS (−)	Acclaim RSLC 120 (2.1 mm ID × 150 mm, 2.2 μm)	A: 0.1% FA; B: ACN; flow rate: 0.4 mL/min	%B (min): 10 (0–2); 95 (17)
*F. distichus* [107,108]	NP-HPLC-DAD-ESI-MS/MS (+)	Develosil Diol (4.6 mm ID × 250 mm, 5 μm)	A: 0.2% HOAc in ACN; B: MeOH:H_2_O:HOAc (97:3:0.2); flow rate: 0.8 mL/min	%B (min): 0 (0); 40 (35); 100 (40); 100 (45)
*A. nodosum* [109]	RP-UHPLC-DAD-ESI-HRMS (−)	Zorbax SB C18 (2.1 mm ID × 100 mm, 1.8 μm)	A: 0.1% FA in MeOH; B: 0.1% FA; flow rate: 0.2 mL/min	%A (min): 3 (50); 70 (50)
*Silvetia compressa* [110]	RP-HPLC-DAD-ESI-MS (+)	Luna C18 (4.6 mm ID × 250 mm, 5 μm)	A: 0.1% FA; B: MeOH; flow rate: 1 mL/min	%B (min): 10 (0–5); 60 (30); 60 (35)
*Sargassum fusiforme* [49]	RP-UHPLC-DAD-ESI-MS/MS (+/−)	Poroshell 120 EC-C18 (2.1 mm ID × 50 mm, 2.7 μm)	A: H_2_O; B: ACN; flow rate: 0.4 mL/min	%B (min): 5 (0–4); 10 (10); 20 (15); 20 (20); 40 (40); 45 (52); 75 (55)
*Sargassum muticum*, *Cystoseira* *abies-marina* [4,92]	HILIC x RP-2D-HPLC-DAD-ESI-MS/MS (−)	D1–Lichrospher diol-5 (1.0 mm ID × 150 mm, 5 μm); D2–Ascentis Express C18 (4.6 mm ID × 50 mm, 2.7 μm)	D1–A: 1% HOAc in ACN; B: 95:3:2 MeOH:H_2_O:HOAc; flow rate: 0.015 mL/min D2–A: 0.1% FA; B: ACN; flow rate: 3 mL/min	D1–%B (min): 0 (0–3); 7 (5); 15 (30); 15 (70); 25 (75); 25 (85) D2–%B (min): 0 (0–0.1); 5 (0.3); 70 (0.8); 90 (0.9)
*Sargassum spinuligerum*, *Cystophora torulosa* [82,83,111,112]	NP-HPLC-DAD	LiChrosorb Si-60 (16 mm ID × 250 mm, 7 μm and 8 mm ID × 250 mm, 5 μm)	A: CHCl_3_; B: EtOH; flow rate: unspecified	Unspecified
*Sargassum wightii* [113]	RP-UHPLC-DAD-ESI-MS (+)	Not specified	A: 0.1% FA; B: ACN; flow rate: 0.3 mL/min	%B (min): 60 (isocratic)
*Cystoseira sauvageauana* [114]	RP-HPLC-DAD-ESI-MS/MS (−)	Eurospher II 100–2 C18 (2.0 mm ID × 250 mm, 2 μm)	A: 5 mM NH_4_CH_3_CO_2_; B: ACN; flow rate: 0.6 mL/min	%B (min): 5 (0–15); 30 (30); 80 (31)
*C. compressa* [115]	RP-UHPLC-DAD-ESI-MS/MS (−)	ACQUITY UPLC-BEH C18 (2.1 mm ID × 50 mm, 1.7 μm)	A: 0.1% FA; B: 0.1% FA in MeOH; flow rate: 0.2 mL/min	Unspecified
*Cystoseira barbata* [116]	RP-UHPLC-DAD-ESI-MS/MS (−)	Zorbax Rapid Resolution High Definition Eclipse Plus C18 (2.1 mm ID × 50 mm, 1.8 μm)	A: 0.1% FA; B: ACN; flow rate: 0.3 mL/min	%B (min): 0.5 (0–1); 30 (7); 95 (8); 95 (10)
*Bifurcaria bifurcata* [117]	RP-HPLC-DAD-ESI-MS/MS (+)	Kromasil 100 C18 (0.46 mm ID × 25 mm, 5 μm)	A: H_2_O; B: EtOH; flow rate: 0.7 mL/min	%B (min): 1 (0–30); 95 (32)

CAN—Acetonitrile; DCM—Dichloromethane; EtOH—Ethanol; MeOH; Methanol; FA—Formic acid; HOAc—Acetic acid; ID—Inner diameter.

**Table 3:** Mass spectrometric data of phlorotannins detected in Fucales.

[M-H]^−/+^ ([M-H]^2−/+^), *m*/*z*	Compound/Group of Compounds	Species (No. Isomers Detected) [Ref]
*DP1*		
125^−^	Phloroglucinol	*H. elongata* (1) [121]; *S. wightii* (1) [122]
*DP2*		
247^−^	Dibenzodioxin-1,3,6,8-tetraol	*A. nodosum* (1) [101], (2) [88]; *F. vesiculosus* (1) [66], (1) [48]
249^−/^251^+^	Difucol/Diphlorethol	*A. nodosum* (2) [88]; *S. fusiforme* (1) [49]
263^−^	Carmalol	*S. fusiforme* (1) [49]
265^−^/267^+^	Bifuhalol	*S. fusiforme* (1) [49]; *C. flexuosum (2)* [100]
273^+^ (+Na)	Diphlorethol	*H. siliquosa* (1) [123]
*DP3*		
369^−^/371^+^	Eckstolonol	*F. guiryi* (1) [16]; *F. spiralis* (1) [16]; *F. vesiculosus* (1) [96]; *S. compressa* (1) [110]; *S. fusiforme* (1) [49]
371^−^	Eckol	*S. fusiforme* (1) [49]
373^−^/375^+^	Fucol/Phlorethol/Fucophlorethol	*C. barbata* (3) [116]; *F. vesiculosus* (1) [16], (3) [105]; *F. serratus* (1) [16]; *F. spiralis* (1) [16]; *C. usneoides* (2) [47]; *D. antarctica* (3) [95]; *F. vesiculosus* (4) [50]; *S. compressa* (4) [110]
373^−^	Trifucol	*F. vesiculosus* [48]
375^+^	Triphlorethol	*H. siliquosa* [123]
389^−^/391^+^	Trifuhalol	*A. nodosum* (1) [44], (1) [79]; *C. flexuosum* (1) [110]; *S. fusiforme* (1) [16]; *S. muticum* (3) [92]
405^−^/407^+^	Hydroxytrifuhalol A	*A. nodosum* (1) [88]; *C. flexuosum* (1) [100]; *S. compressa* (1) [110]
387^−^	7-Hydroxyeckol	*A. nodosum* (1) [88]
387^−^	Phlorethoxycarmalol	*S. fusiforme* (1) [49]
413^+^ (+Na)	Trifuhalol	*H. siliquosa* (1) [123]
*DP4*		
493^−^	Hydroxyfucofuroeckol	*F. guiryi* (1) [16]
479^−^	Fucofurodiphlorethol	*F. vesiculosus* (1) [66], (2) [48], (1) [96]
495^−^/497^+^	Phloroeckol	*C. tamariscifolia* (1) [47]; *C. nodicaulis* (2) [47]; *D. antarctica* (1) [95]; *A. nodosum* (2) [124]
497^+^	7-Phloroeckol	*S. compressa* (1) [110]
497^−^/499^+^	Fucol/Phlorethol/Fucophlorethol	*C. barbata* (4) [116]; *F. spiralis* (1) [47], (2) [16]; *F. guiryi* (5) [16]; *A. nodosum* (1) [44], (1) [88]; *F. serratus* (3) [16]; *D. antarctica* (1) [95]; *C. nodicaulis* (3) [47]; *C. tamariscifolia* (1) [47]; *C. usneoides* (1) [47]; *F. vesiculosus*(4) [50], (2) [106], (2) [16]
497^−^	Tetrafucol	*F. vesiculosus* (2) [66], (1) [48], (1) [46]
497^−^	Fucophlorethol	*F. vesiculosus* (1) [46]
511^−^	Diphlorethohydroxycarmalol	*A. nodosum* (1) [101]; *F. vesiculosus* (1) [46], *S. fusiforme* (1) [49]
513^−^/515^+^	Tetrafuhalol	*C. flexuosum* (1) [100]; *A. nodosum* (1) [79]; *B. bifurcata* (1) [79]; *S. fusiforme* (1) [49]; *S. muticum* (5) [93]
527^−^	Hydroxydiphlorethoxycarmalol	*S. fusiforme* (1) [49]
529^−^/531^+^	Hydroxytetrafuhalol	*F. vesiculosus* (1) [66]; *C. flexuosum* (2) [101]; *F. vesiculosus* (1) [48], (1) [79], (1) [46]; *B. bifurcata* (1) [79]; *S. fusiforme* (1) [49]; *S. muticum* (6) [92]
545^−^/547^+^	Dihydroxytetrafuhalol	*B. bifurcata* (1) [79]; *F. vesiculosus* (1) [79]; *S. compressa* (1) [110]; *S. muticum* (1) [92]
537^+^ (+Na)	Tetrafuhalol	*H. siliquosa* (1) [123]
591^−^	Diphlorethohydroxycarmalol sulphate	*A. nodosum* (1) [101]
*DP5*		
601^−^/603^+^	Phlorofucofuroeckol	*C. barbata* (1) [116]; *C. tamariscifolia* (2) [47]
603^+^	Phlorofucofuroeckol A	*S. compressa* (1) [110]
603^−^	Fucofurotriphlorethol	*F. vesiculosus* (1) [48]; *F. vesiculosus* (1) [66]
621^−^/623^+^	Fucol/Phlorethol/Fucophlorethol	*C. barbata* (4) [116]; *C. usneoides* (1) [47]; *C. abies-marina* (8) [4]; *D. antarctica* (4) [96]; *A. nodosum* (1) [44]; *S. fusiforme* (1) [49]; *F. vesiculosus* (4) [50], (7) [16], (3) [105];
621^−^	Pentafucol	*F. vesiculosus* (1) [66]
621^−^	Trifucophlorethol	*F. vesiculosus* (1) [48], (1) [96],
621^−^	Pentaphlorethol	*S. muticum* (1) [92]
635^−^	Triphlorethoxycarmalol	*S. fusiforme* (1) [49]
637^−^	Pentafuhalol	*F. vesiculosus* (1) [66], (2) [48]; *A. nodosum* (1) [79], *S. fusiforme* (1) [49], *S. muticum* (5) [92]
639^+^	Pentafuhalol	*S. compressa* (1) [110]
651^−^	Hydroxytriphlorethoxycarmalol	*S. fusiforme* (1) [49]
653^−^	Hydroxypentafuhalol	*S. muticum* (2) [92]; *S. fusiforme* (1) [49]
669^−^	Dihydroxypentafuhalol	*S. muticum* (3) [92]
671^+^	Dihydroxypentafuhalol	*S. compressa* (1) [110]
*DP6*		
741^−^/743^+^	Dieckol	*C. tamariscifolia* (1) [47]; *S. compressa* (1) [110]; *S. fusiforme* (1) [49]
745^−^/747^+^	Fucol/Phlorethol/Fucophlorethol	*D. antarctica* (6) [95]; *C. abies-marina* (2) [4]; *C. barbata* (3) [116]; *F. spiralis* (1) [47]; *A. nodosum* (1) [44]; *F. vesiculosus* (3) [50], (2) [16], (3) [105]; *S. fusiforme* (1) [49]
745^−^	Hexafucol	*F. vesiculosus* (1) [66], (1) [48], (1) [96]
745^−^	Tetrafucophlorethol	*F. vesiculosus* (1) [96]
745^−^	Hexaphlorethol	*S. muticum* (1) [92]
759^−^	Tetraphlorethoxycarmalol	*S. fusiforme* (1) [49]
761^−^	Hexafuhalol	*S. fusiforme* (1) [49]; *S. muticum* (3) [92]
777^−^/779^+^	Hydroxyhexafuhalol	*C. flexuosum* [100]; *F. vesiculosus* (2) [79]; *S. fusiforme* (1) [49]; *S. muticum* (2) [92]
791^−^	Dihydroxytetraphlorethoxycarmalol	*S. fusiforme* (1) [49]
793^−^	Dihydroxyhexafuhalol	*S. fusiforme* (1) [49]; *C. flexuosum* (3) [100]; *S. muticum* (6) [92]
809^−^	Trihydroxyhexafuhalol	*S. muticum* (1) [92]
*DP7*		
851^−^	Fucofuropentaphlorethol	*F. vesiculosus* (1) [48]
869^−^/871^+^	Fucol/Phlorethol/Fucophlorethol	*D. antarctica* (3) [95]; *C. abies-marina* (4) [4]; *C. barbata* (3) [116]; *F. spiralis* (3) [47]; *F. vesiculosus* (2) [50], (2) [105]
869^−^	Heptafucol	*F. vesiculosus* (1) [66]
869^−^	Trifucotriphlorethol	*F. vesiculosus* (1) [48]
869^−^	Difucotetraphlorethol	*F. vesiculosus* (1) [48]
869^−^	Heptaphlorethol	*S. muticum* (1) [92]
883^−^	Pentaphlorethoxycarmalol	*S. fusiforme* (1) [49]
887^−^	Heptafuhalol	*S. fusiforme* (1) [49]
901^−^	Hydroxyheptafuhalol	*S. muticum* (1) [93]; *S. fusiforme* (1) [49]
915^−^	Dihydroxypentaphlorethoxycarmalol	*S. fusiforme* (1) [49]
917^−^	Dihydroxyheptafuhalol	*S. fusiforme* (1) [49]; *S. muticum* (5) [92]
933^−^	Trihydroxyheptafuhalol	*B. bifurcata* (1) [79]; *S. muticum* (2) [92]
*DP8*		
993^−^/995^+^	Fucol/Phlorethol/Fucophlorethol	*D. antarctica* (3) [95]; *C. abies-marina* (4) [4]; *F. spiralis* (3) [47]; *A. nodosum* (1) [79]; *S. fusiforme* (1) [49]
993^−^	Pentafucodiphlorethol	*F. vesiculosus* (1) [48]
993^−^	Hexafucophlorethol	*F. vesiculosus* (1) [48]
993^−^	Tetrafucotetraphlorethol	*F. vesiculosus* (2) [48]
1007^−^	Hexaphlorethoxycarmalol	*S. fusiforme* (1) [49]
1009^−^	Octafuhalol	*S. muticum* (1) [92]
1041^−^	Dihydroxynonafuhalol	*S. fusiforme* (1) [49]; *S. muticum* (1) [92]
1043^+^	Hydroxyoctafuhalol	*C. flexuosum* (1) [100]
1055^−^	Trihydroxyhexaphlorethoxycarmalol	*S. fusiforme* (1) [49]
1057^−^	Trihydroxyoctafuhalol	*S. fusiforme* (1) [49]; *S. muticum* (2) [92]
*DP9*		
1117^−^/1119^+^	Fucol/Phlorethol/Fucophlorethol	*C. abies-marina* (4) [4]; *F. vesiculosus* (3) [50]; *F. distichus* (1) [108]; *S. fusiforme* (1) [49]
1133^−^	Nonafuhalol	*S. muticum* (2) [92]
1165^−^	Dihydroxinonafuhalol	*S. fusiforme* (1) [49]; *S. muticum* (1) [93]
1181^−^	Trihydroxynonafuhalol	*S. fusiforme* (1) [49]
*DP10*		
1241^−^/1243^+^	Fucol/Phlorethol/Fucophlorethol	*C. abies-marina* (4) [4]; *F. vesiculosus* (2) [50]; *A. nodosum* (2) [122]; *F. distichus* (1) [108]; *S. fusiforme* (1) [49]
1257^−^	Decafuhalol	*S. muticum* (1) [92]
1305^−^	Trihydroxydecafuhalol	*S. fusiforme* (1) [49]
1321^−^	Tetrahydroxydecafuhalol	*S. fusiforme* (1) [49]
*DP11*		
1365^−^	Fucol/Phlorethol/Fucophlorethol	*C. abies-marina* (4) [4]; *A. nodosum* (1) [124]; *F. distichus* (1) [108]; *S. fusiforme* (1) [49]
1445^−^	Tetrahydroxyfuhalol	*S. fusiforme* (1) [49]
*DP12*		
1489^−^ (744^−^)	Fucol/Phlorethol/Fucophlorethol	*C. abies-marina* (3) [4]; *F. distichus* (1) [108]
1585^−^	Pentahydroxyfuhalol	*S. fusiforme* (1) [49]
*DP13*		
(806^−^)	Fucol/Phlorethol/Fucophlorethol	*C. abies-marina* (2) [4]
(806^−^)	Fucol/Phlorethol/Fucophlorethol	*F. distichus* (1) [108]
*DP14*		
1737^−^ (868^−^)	Fucol/Phlorethol/Fucophlorethol	*C. abies-marina* (2) [4]; *A. nodosum* (2) [124]; *F. distichus* (1) [108]
*DP15*		
(930^−^)	Fucol/Phlorethol/Fucophlorethol	*C. abies-marina* (1) [4]; *F. distichus* (1) [108]
*DP16*		
(992^−^)	Fucol/Phlorethol/Fucophlorethol	*C. abies-marina* (4) [4]; *F. distichus* (1) [108]
*DP17*		
(1054^−^)	Fucol/Phlorethol/Fucophlorethol	*C. abies-marina* (1) [4]; *F. distichus* (1) [108]
*DP18*		
(1116^−^)	Fucol/Phlorethol/Fucophlorethol	*F. distichus* (1) [108]
*DP19*		
(1178^−^)	Fucol/Phlorethol/Fucophlorethol	*F. distichus* (1) [108]
*DP20*		
(1240^−^)	Fucol/Phlorethol/Fucophlorethol	*F. distichus* (1) [108]

**Table 4:** Typical ^13^C NMR signals associated with phlorotannin structural identification.

δ (ppm)	Bound Type	Conclusion
100	-C-C-	-
120–150	-C-O-	Presence of phlorethols
145–155	Signals for hydroxy groups additional to the monomer -OH	Presence of fuhalols
166–168	-C=O	From the acetyl groups

**Table 5:** Examples of phlorotannins (groups or compounds) identified using NMR spectroscopy in distinct Fucales.

Species	Identified Phlorotanins Using NMR	Ref.
*Cystoseira tamariscifolia*	Fuhalols: bifuhalol hexaacetate Phlorethols: diphlorethol pentaacetate	[136]
*Fucus vesiculosus*	Fucophlorethols: trifucotriphlorethol A, fucotriphlorethol E Fucols: tetrafucol A, tetrafucol B	[138]
*Fucus spiralis*	Groups fucol and fucophlorethol	[137]
*Bifurcaria bifurcata*	Fuhalols: tetrafuhalol-B-undecacetate, desacetoxyheptafuhalol- heptadecacetate, heptafuhaloloctadecacetate, nonafuhaloltricosacetate, undecafuhaloloctacosacetate, tridecafuhaloltritriacontacetate	[136]
*Halidrys siliquosa*	Fuhalols: trifuhalol, tetrafuhalol Phlorethols: diphlorethol, triphlorethol	[139]
*Sargassum spinuligerum*	Fuhalols: hydroxytrifuhalol-A nonaacetate, hydroxytrifuhalol-B nonaacetate, hydroxypentafuhalol-B tetradecaacetate, deshydroxytetrafuhalol-B decaacetate, deshydroxyhexafuhalol-B pentadecaacetate, hydroxyheptafuhalol-A nonadecaacetate, hydroxyheptafuhalol-B nonadecaacetate, pentafuhalol-A tridecaacetate, heptafuhalol-A octadecaacetate, pentafuhalol-B tridecaacetate, heptafuhalol-B octadecaacetate, nonafuhalol-B tricosaacetate, trifuhalol-B octaacetate, tetrafuhalol-B undecaacetate, hexafuhalol-B hexadecaacetate Phlorethols: diphlorethol, triphlorethol, diphloretholpentaacetate, triphlorethol-A heptaacetate, triphlorethol- B heptaacetate, tetraphlorethol-C nonaacetate, dihydroxytetraphlorethol-A undecaacetate, dihydroxytetraphlorethol-B undecaacetate, pentaphlorethol-A undecaacetate Fucophlorethols: fucophlorethol-B octaacetate, fucodiphlorethol-B decaacetate, fucodiphlorethol-D decaacetate, fucodiphlorethol-F decaacetate, bisfucotriphlorethol-A pentadecaacetate, bisfucotetraphlorethol-A heptadecaacetate, chlorobisfucopentaphlorethol nonadecaacetate, difucodiphlorethol-A tridecaacetate, fucodifucotetraphlorethol-A icosaacetate Hydroxyfucophlorethols: hydroxyfucophlorethol-A undecaacetate, hydroxybisfucophlorethol-A hexadecaacetate, dihydroxyfucotriphlorethol-A tetradecaacetate, dihydroxyfucotriphlorethol-B tetradecaacetate, bisfucopentaphlorethol-B nonadecaacetate, chlorobisfucopentaphlorethol-A nonadecaacetate	[82,83]
*Cystophora torulosa*	Fucols: difucol hexaacetate, trifucol nonaacetate Phlorethols: diphlorethol pentaacetate, triphlorethol-A- heptaacetate, chlorobisfucopentaphlorethol-A nonadeca-acetate Fuhalols: trifuhalol-A octaacetate, tetrafuhalol-A undecaacetate, pentafuhalol-A tridecaacetate, hexafuhalol-A hexadecaacetate Fucophlorethols: fucophlorethol-B octaacetate, fucophlorethol-B decaacetate, fucodiphlorethol-D decaacetate, difucophlorethol-A undecaacetate, fucotriphlorethol-B dodecaacetate, fucotetraphlorethol-B tetradecaacetate, bisfucotriphlorethol-A pentadecaacetate, bisfucotetraphlorethol-A heptadecaacetate, bisfucopentaphlorethol-A nonadecaacetate, fucophlorethol-C octaacetate, fucodiphlorethol-E decaacetate, fucodifucotetraphlorethol-A eicosaacetate, bisfucotriphlorethol-B pentadecaacetate Hydroxyfucophlorethols: hydroxyfucophlorethol-A nonaacetate, hydroxyfucodiphlorethol-A undecaacetate, hydroxyfucotriphlorethol-A tridecaacetate, hydroxyfucotetraphlorethol-A pentadecaacetate, hydroxyfucopentaphlorethol-A heptadecaacetate, hihydroxyfucotriphlorethol-A tetradecaacetate, hihydroxyfucotetraphlorethol-A hexadecaacetate, trihydroxyfucopentaphlorethol-A nonadecaacetate, hydroxyfucodiphlorethol-B undecaacetate, hydroxyfucotriphlorethol-B tridecaacetate, hydroxybisfucopentaphlorethol-A eicosaacetate	[135]
